# Seven-year follow-up of a patient with hereditary gingival fibromatosis treated with a multidisciplinary approach: case report

**DOI:** 10.1186/s12903-021-01830-7

**Published:** 2021-09-26

**Authors:** Ning Li, Wenfang Wang, Yuanyuan Sun, Hongning Wang, Tiejun Wang

**Affiliations:** 1 Department of Orthodontics, Yantai Stomatological Hospital, Yantai, 264000 Shandong China; 2 Department of Stomatology, The First Affiliated Hospital of Xi’an Jiaotong University, Xi’an, 710000 Shanxi China; 3 Department of Periodontology, Yantai Stomatological Hospital, Yantai, 264000 Shandong China

**Keywords:** hereditary gingival fibromatosis (HGF), Orthodontic, Plaque, Case report

## Abstract

**Background:**

Hereditary gingival fibromatosis (HGF) is rare in clinical practice, and the long-term results of the combined orthodontic-periodontal treatment of HGF are rarely reported.

**Case presentation:**

This study reports for the first time the results of seven years of follow-up in a seven-year-old girl with HGF. The diagnosis was confirmed by clinical signs, family history and histopathological examination. First, periodontal scaling and oral hygiene reinforcement were performed regularly in the mixed dentition stage. Next, gingivoplasty was performed on the permanent dentition. Two months after the surgery, treatment with fixed orthodontic appliances was conducted. The teeth were polished on a monthly basis, and oral hygiene was reinforced to control gingival enlargement. Gingival hypertrophy recurred slightly, and gingivectomies were performed in the months following the start of orthodontic treatment. Follow-up was performed for 24 months with orthodontic retention, and gingival enlargement remained stable after the combined treatment.

**Conclusions:**

The risk of gingival hyperplasia recurrence during and after orthodontic treatment is high, but satisfying long-term outcomes can be achieved with gingivectomy, malocclusion correction, and regular follow-up maintenance.

## Background

Hereditary gingival fibromatosis (HGF) is a rare, hereditary, benign disorder characterized by slow and progressive fibrous hyperplasia of the gingiva. The prevalence of this condition is low (1/175,000 inhabitants) [1]. HGF normally appears with the eruption of permanent teeth, although cases have been described in patients with primary teeth and even at birth. Gingival enlargement may occur alone or in conjunction with other abnormalities, such as part of a syndrome, and is most commonly associated with hypertrichosis and epilepsy, with or without mental retardation [2].

The aetiology and pathogenesis of HGF are unclear. HGF is an autosomal dominant genetic disorder [3, 4] with a high degree of genetic heterogeneity [5]. However, an autosomal recessive mode of inheritance has also occasionally been reported [6]. The pathogenesis of the disease is based on connective tissue defects due to gene mutations [7–9]. In addition, sex hormones and epidermal growth factor also play a role in the abnormal proliferation of gingival fibres [10]. Clinically, HGF can lead to malocclusion, delayed eruption of permanent teeth, and speech, articulation and mastication disorders, which can have a negative aesthetic and psychological impact on patients. Without intervention, periodontal disease will develop [11, 12]. Currently, all treatments for HGF are invasive. Gingivoplasty is the main clinical treatment, with the possibility of recurrence [13]. In this paper, we report the case of a girl with HGF whose periodontal tissue and dentition showed satisfactory improvement after hybrid periodontal-orthodontic treatment and seven years of follow-up.

### Case presentation

**Fig. 1:**
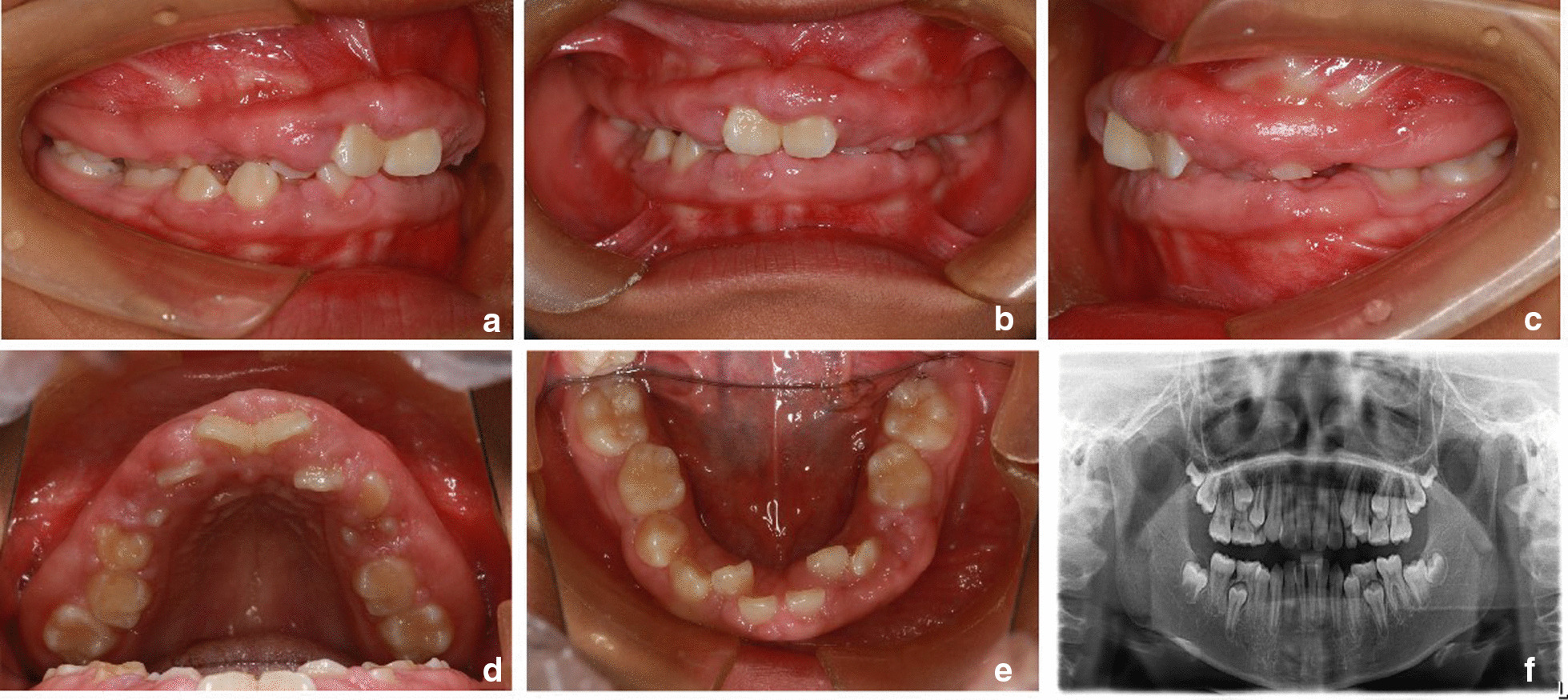
Pantomogram of the primary dentition (**a**–**e**) during the first visit (patient age, seven years)

The patient was female and was seven years old when she first visited the orthodontics department for non-eruption of the upper teeth and gingival enlargement. The patient's mother also had a history of similar gingival enlargement. There was no history of taking medication. No deformities of the spine or limbs were observed, and normal nail development and normal hair growth on the face were observed. The intraoral examination revealed average oral hygiene with a small amount of plaque on the surface of the teeth. Both the free and attached gingiva showed diffuse, irregular hyperplasia and hypertrophy in the upper and lower mixed dentition (Fig. 1a–e). Bleeding on probing (BOP) was present in all areas, with a gingival index (GI) of 2 [14]. The periodontal probing depth ranged from 4 to 8 mm. The pantomogram revealed no significant resorption of the alveolar bone and the absence of anomalies in tooth number (Fig. 1f), as well as permanent dentition with retained deciduous molars with stage 3 to 5 root development [15]. The patient was diagnosed with HGF by a periodontist. Considering that the patient had mixed dentition, the periodontist recommended basic periodontal therapy, such as teeth polishing, scaling and oral hygiene reinforcement, as well as follow-up of tooth eruption. During this period, periodontal maintenance therapy was performed every six months.

### Diagnostic features at the time of presentation prior to the start of orthodontic treatment

**Fig. 2:**
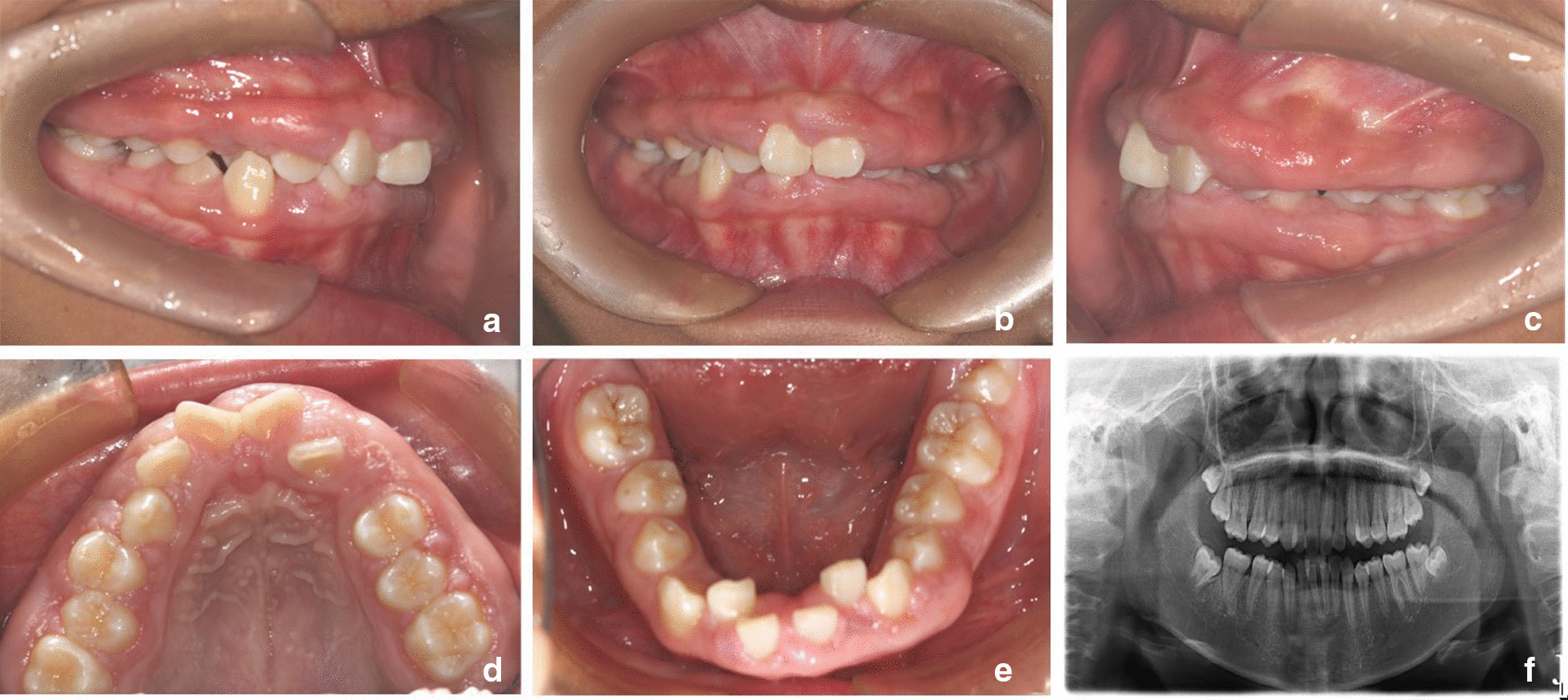
**a**–**f** Patient follow-up, two years after the first visit

The patient underwent a follow-up examination two years later. The extraoral examination revealed an asymmetrical face, resulting in deviation of the chin to the left. The upper and lower lips were protruding. The intraoral examination showed narrowing of the upper and lower permanent dentition, with a grade 3 overjet and a grade 1 overbite. The oral hygiene was normal, with a GI of two [16]. A small amount of subgingival calculus was detected upon exploration, with a periodontal pocket depth of 4–8 mm and a loss of attachment of 0–1 mm. There was still extensive gingival hyperplasia in the upper teeth, affecting the free gingiva, attached gingiva and gingival papillae. The pantomogram showed no significant resorption of the alveolar bone, and the roots were well developed (Fig. 2). The clinical diagnosis was HGF (angle class II, skeletal class I, and medium angle). Treatment included serial basic periodontal treatment, surgical periodontal treatment, periodontal maintenance therapy and orthodontic treatment. The case study was conducted with the parents’ understanding and written consent for the intervention and research participation. Also, the study was approved by the Research Ethics Committee of Yantai Stomatological Hospital.

### Sequence of the periodontal treatment

For the first month, basic periodontal therapy and oral hygiene education were performed. Unfortunately, no significant improvement in the gingival tissue was seen. Next, periodontal surgery was performed. After iodophor disinfection and local infiltration anaesthesia with articaine and epinephrine, the depth of gingival hyperplasia was assessed with a periodontal probe, and the excessive gingiva was removed. A successive, external bevel incision was made using a conventional scalpel. After removal, the gingiva was scalloped in shape, with slightly thinner margins. The gingival papillae were also sectioned. Then, a periodontal curette was used to remove any remaining calculus, pathological granulation tissue and diseased cementum from the root surface. Postoperatively, the removed gingival tissue was examined pathologically. The patient was advised to continue taking the antibiotic (amoxicillin, 500 mg tds) for 3 days. A 0.12% chlorhexidine gluconate rinse was prescribed for administration twice a day for one week. Additionally, the patient was taught to brush her teeth regularly, and oral hygiene was reinforced.

### Histopathological examination

**Fig. 3:**
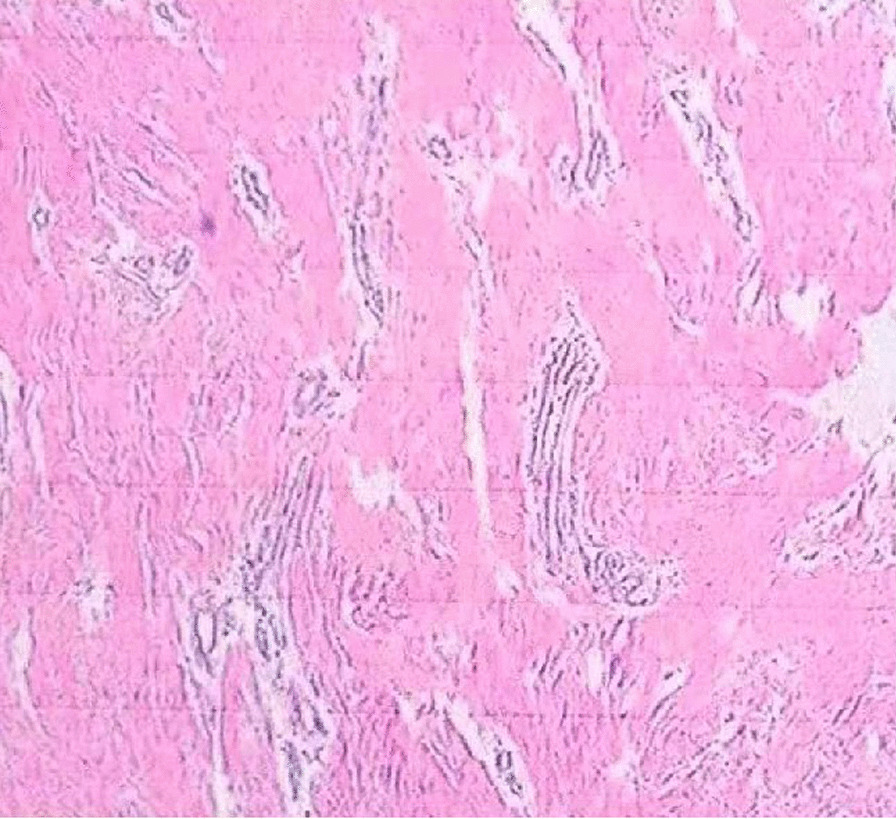
Histopathological examination

After haematoxylin and eosin (H&E) staining, a routine pathological examination of the hyperplastic gingival tissues showed a mature fibrous tissue composition with high levels of collagen fibres and focal inflammatory cell infiltration, consistent with the characteristics of HGF (Fig. 3).

### Periodontal and orthodontic management

**Fig. 4:**
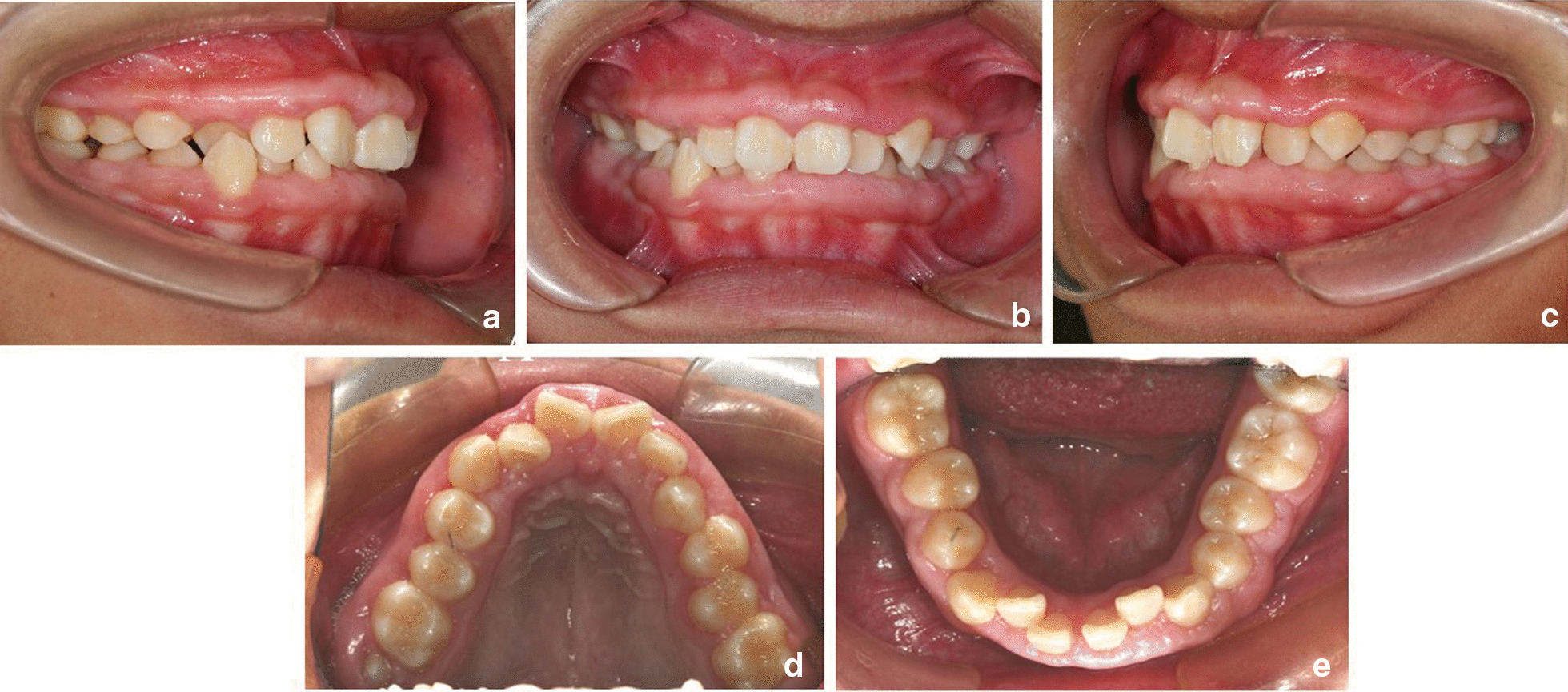
**a**–**e** Two months after periodontal surgery and before the start of orthodontic treatment

**Fig. 5:**
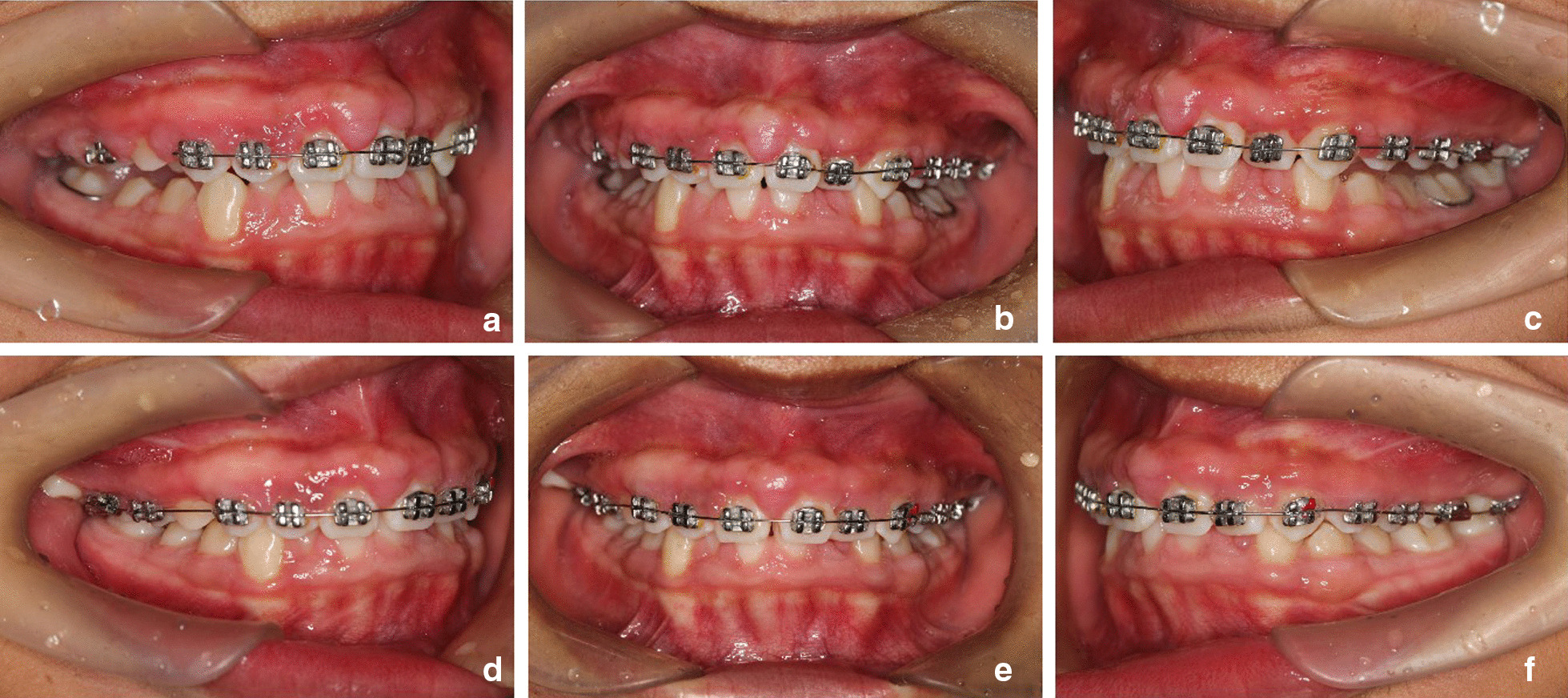
Gingival redness and hyperplasia one year after orthodontic treatment (**a**–**c**); during treatment, one month after the second gingivectomy (**d**–**f**)

Two months after the gingivectomy, the patient was able to maintain good oral hygiene, and the crowns adequately exposed. Moreover, the gingival morphology in the upper dentition significantly improved, while both the BOP percentage and probing depth (PD) were significantly reduced (Fig. 4a–e). After a comprehensive assessment, orthodontic treatment with fixed appliances began. The upper teeth were treated first due to the deep overjet of the anterior teeth. Periodontal follow-up was performed every month, along with the polishing of all teeth and oral hygiene reinforcement. However, one year after orthodontic treatment, gingival enlargement was found in the maxillary premolar and molar areas after placement of the brackets (Fig. 5a–c). Because the patient's dental crowns were so small, it was difficult to remove plaque in these areas. The gingival enlargement showed improvement one month after the second gingivectomy (Fig. 5d–f). Once the overjet was corrected, orthodontic treatment was conducted on the lower teeth. Considering that the lower teeth were more vulnerable to plaque build-up, periodontal maintenance therapy was carried out throughout the orthodontic treatment, and the patient was instructed to visit the periodontal department every month for regular teeth polishing and oral hygiene reinforcement.

**Fig. 6:**
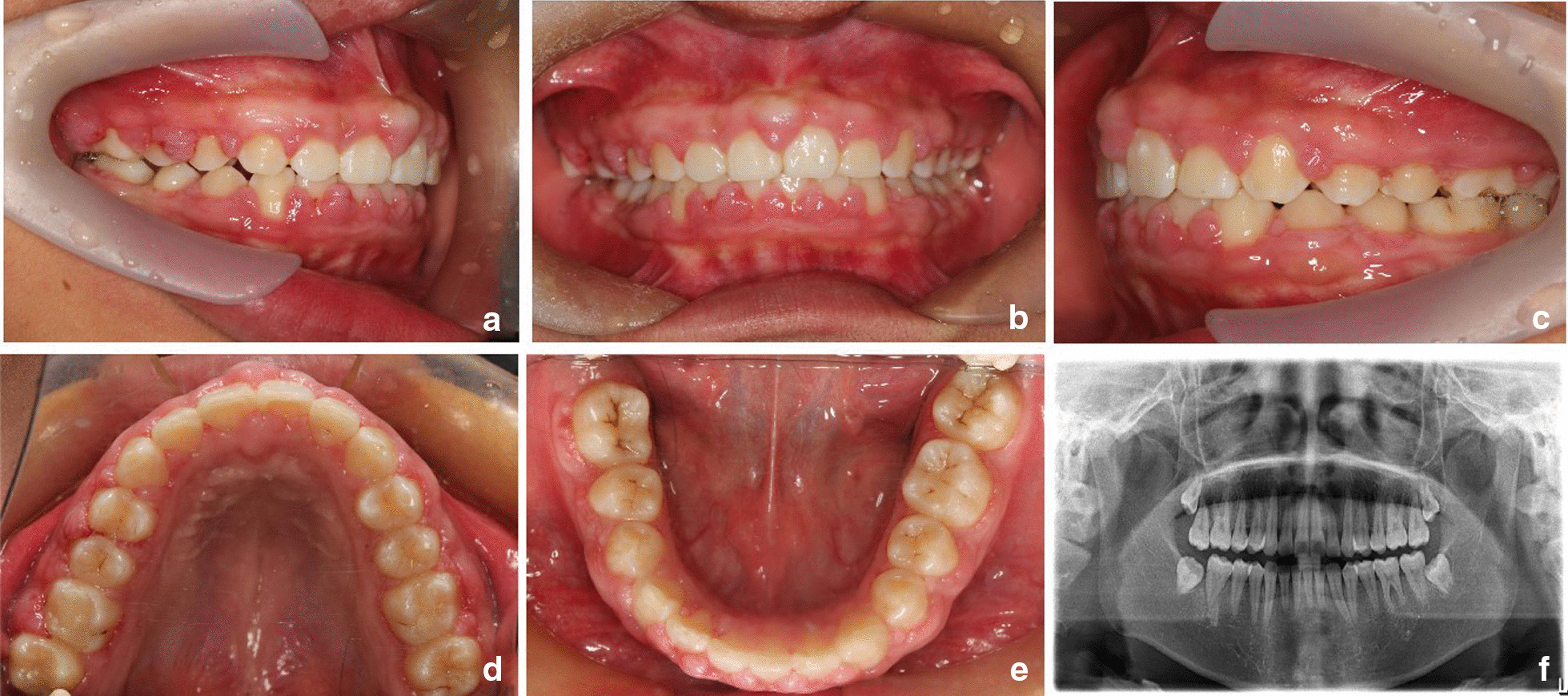
**a**–**f** Completion of the orthodontic treatment

The orthodontic treatment lasted for three years. After treatment, the upper and lower dentition were aligned. The deep overjet and overbite as well as the occlusion of the anterior teeth were improved. The patient's periodontal condition was well managed, with gingival hyperplasia only in the right maxillary molar area and the anterior mandibular region (Fig. 6a–e). The pantomogram revealed no significant resorption of the alveolar bone (Fig. 6f). To achieve better oral hygiene, removable Hawley retainers were used instead of lingual fixed retainers or vacuum-formed retainers. Gingivectomies were performed in the months following the start of retention. Periodontal follow-up was performed every 3 months, along with the polishing, scaling and oral hygiene reinforcement each time. Over 24 months of follow-up after the completion of orthodontic treatment, gingival hypertrophy recurred slightly in the maxillary molar areas. The periodontal PD ranged from 3 to 5 mm and scaling was used to remove the subgingival calculus. Since then, the patient has not returned periodically for observation because she went to college in another city, increasing the difficulty of follow-up.

## Discussion

Currently, long-term, progressive treatment is suggested as the first-line treatment for HGF, involving periodontal maintenance, surgery, orthodontic treatment and tooth extraction [17–21]. The surgical treatment of choice often includes gingivectomy, electrocautery and carbon dioxide lasers [11, 13]. In this study, the patient had crowded teeth with poor occlusion, which increased the chance of plaque build-up and was detrimental to long-term periodontal maintenance. Thus, regular follow-up and basic periodontal treatment were performed on the mixed dentition of this patient. On the permanent dentition, we performed an external bevel gingivectomy to improve the gingival morphology. Postoperatively, orthodontic treatment was conducted to align the teeth and achieve better oral hygiene and long-term periodontal stability. The patient's periodontal condition improved significantly after the combined orthodontic and periodontal treatment, and the orthodontic treatment did not result in any increased loss of periodontal attachment.

Regarding the timing of HGF treatment, some researchers recommend early surgery [11]. While this approach has a positive effect on a child’s psychological development and is effective in correcting malocclusion, retention of the primary teeth, and aesthetic and mastication issues, its recurrence rate is high [22]. Most studies have found that the best timing for surgery is when all permanent teeth have erupted, and the upper and lower jaws are well developed [13]. Oral hygiene also plays an important role in the recurrence of HGF [23]. Therefore, it is important to conduct basic periodontal therapy, plaque and calculus removal, and oral hygiene maintenance either before, during, or after surgery. Based on clinical experience, we suggest that the timing of the treatment should be determined by the clinical eruption of the permanent teeth. If HGF has affected the eruption of the permanent teeth, resulting in delayed or ectopic eruption, early surgery should be conducted to facilitate eruption. Otherwise, gingivectomy can be performed after the permanent teeth have erupted. In this case, we performed gingivectomy before orthodontic treatment of the permanent dentition. After surgery, the gingiva had an improved shape, and the patient's chewing and lip closure had returned to normal. In addition, little gingival enlargement was found during the post-orthodontic treatment follow-up.

Orthodontic treatment in patients with HGF should be performed after periodontal inflammation has been controlled, which is approximately two months after basic periodontal therapy and surgery. In addition, orthodontic treatment may exacerbate the patient's gingival hyperplasia [2], which requires particular attention during periodontal maintenance. After orthodontic treatment, follow-up examinations were conducted every three weeks, at which point the periodontal status was assessed. Periodontal scaling was performed every 2–3 months, and gingivectomy was performed a second time for teeth with severe gingival hyperplasia. It is therefore important to conduct oral hygiene maintenance and regular periodontal follow-up during orthodontic treatment to prevent the recurrence of HGF.

In most cases, orthodontic treatment takes a longer time in patients with HGF due to the thick gingiva encountered in these patients, which acts as a physical barrier to tooth movement [24]. In this study, we found that the patient’s teeth moved at a normal rate during treatment, and no significant loosening was observed. However, gingival hyperplasia and maintenance extended the overall orthodontic treatment timeframe.

HGF is likely to recur, with an overall recurrence rate of 34.92% after surgical treatment [13]. The recurrence rate is related to age, surgical technique and operation, location of hyperplasia, and genetics [25]. Thus, it is necessary to maintain a normal gingival status through multiple surgeries and postoperative plaque control. Additionally, long-term retention after orthodontic treatment is required to maintain stable outcomes, prevent the recurrence of hyperplasia, and consolidate the gingival remodelling process. After orthodontic treatment, the patient should undergo a regular periodontal assessment and basic maintenance therapy every 3 to 6 months.

In this study, satisfactory long-term results were achieved as a result of regular follow-up examinations, a precise clinical diagnosis, and a multidisciplinary treatment combining periodontal, orthodontic and maintenance treatment of the patient with HGF. Although the timing, frequency and volume of surgical procedures for HGF are still controversial, it can be concluded that orthodontic treatment is beneficial for oral hygiene maintenance and gingival stability in HGF patients. In addition, long-term follow-up and maintenance are required to achieve stable periodontal outcomes.

## Data Availability

Not applicable.

## Abbreviation

HGF: Hereditary gingival fibromatosis
